# Construction of a HOXA11-AS-Interacted Network in Keloid Fibroblasts Using Integrated Bioinformatic Analysis and *in Vitro* Validation

**DOI:** 10.3389/fgene.2022.844198

**Published:** 2022-03-31

**Authors:** Qiang Wang, Wei Wang, Xiao-jie Sun

**Affiliations:** ^1^ Department of Obstetrics and Gynecology, The Second Hospital of Jilin University, Changchun, China; ^2^ Department of Radiology, The First Hospital of Jilin University, Changchun, China; ^3^ Department of Plastic Surgery, China-Japan Union Hospital of Jilin University, Changchun, China

**Keywords:** HOXA11-AS, competing endogenous RNA, keloid fibroblast, validation, integrated molecular network

## Abstract

**Background:** Expression of the long noncoding RNA (lncRNA) HOXA11-AS significantly increased in keloids by unclarified molecular regulation mechanisms.

**Methods:** Using successfully primary cultured keloid-derived fibroblasts from central region of chronic keloid tissues (sample 0), small interfering RNAs were designed and transfected into two keloid fibroblast samples (samples 1 and 2) to knockdown HOXA11-AS. One nonspecific transfection control (sample 3) and one blank control (sample 4) were used to remove nonspecific overlap from the studied group. The lncRNAs, messenger RNAs (mRNAs), and microRNAs (miRNAs) of five samples were sequenced to identify differentially expressed (DE) profiles in HOXA11-AS-knockdown keloid fibroblasts in samples 1 and 2 (by intersection), which facilitated removal of overlap with the nonspecific controls (samples 3 and 4, by union). Using stepwise bioinformatic analysis, a HOXA11-AS-interacted competing endogenous network (ceRNA) was screened based on three DE profiles.

**Results:** Keloid fibroblasts with or without HOXA11-AS as well as with or without nonspecific interferences were successfully constructed respectively. A total of 1,396 mRNAs and 39 lncRNAs were significantly changed in keloid fibroblast with HOXA11-AS knockdown. Simultaneously, 1,626 mRNAs and 99 lncRNAs were significantly changed in keloid fibroblast with nonspecific interference. With removal of nonspecific overlap, a lncRNA–mRNA interactive network characterized by close natural/intronic antisense relationship was initially constructed in keloid fibroblast with HOXA11-AS knockdown. Based on this network, a lncRNA–mRNA–protein interaction network was extended by integration of the human protein–protein interaction network. Significant functional genes were screened using PageRank algorithm in the extended network. Three genes, including SNED1, NIPAL3, and VTN, were validated by real-time PCR in HOXA11-AS-knockdown keloid fibroblasts. Only NIPAL3 was predicted to be a target gene for HOXA11-AS *via* three competing endogenous miRNAs (hsa-miRNA-19a-3p, hsa-miR-141-3p, and hsa-miR-140-5p).

**Conclusion:** An interactive network of HOXA11-AS–three miRNAs–NIPAL3 was predicted in keloid fibroblasts by integrative bioinformatic analysis and *in vitro* validation.

## Introduction

Keloids are aberrantly proliferative benign tumors with markedly excessive fibroblast deposition and extracellular matrix extending beyond the normal boundaries during wound healing. Wound healing underwent a dynamic series of pathological changes, forming a highly heterogeneous keloid tissues in predisposed human skins. Lacking of animal models posed a major challenge on molecular pathogenesis of keloid formation (Supp, 2019). Highly heterogeneous cell compositions within a keloid tissue are also difficult to study (Tucci-Viegas et al., 2010; Limandjaja et al., 2018; Limandjaja et al., 2020) even by *in vitro* reconstructed 3D cell cultures (Suttho et al., 2017). Moreover, a paradoxical coexistence of both scar-like (Tan et al., 2019) transformation and cancer-like bioenergetics (Vincent et al., 2008) was shown within a keloid. Such heterogenous compositions and dynamic developments indicate some intensive molecular regulations might exist internally during keloids formation corresponding to healing. As the main cellular components in keloid tissues, keloid-derived fibroblasts served as an overacted regulator for the excessive extracellular matrix remodeling (Luo et al., 2001), however, the central region representing for the well-transformed keloid fibroblasts were difficult to culture and passage due to its severe ischemic status (Touchi et al., 2016). In the peripheral regions of keloids, it was difficult to identify the proportion of transitional fibroblasts from normal to transformed status. Based on such heterogeneity, it was understudied the molecular mechanism of keloid formation.

Using *in vivo* profiling analysis, it showed numerous encoding messenger RNAs (mRNAs), long noncoding RNAs (lncRNAs), and microRNAs (miRNAs) heterogeneously altered in keloid formation (Deng et al., 2020; Duan et al., 2020; Li et al., 2021). Our team had found that HOXA11 antisense RNA (HOXA11-AS) aberrantly upregulated in keloids by comparing with peripheral normal skins (Sun et al., 2017). In keloid-derived fibroblasts, HOXA11-AS was shown to regulate cell proliferation, apoptosis and/or migration *via* miR-124-3p (Jin et al., 2020), miR-205-5p (Su et al., 2021) or *via* miR-148b-3 (Wang and Shen, 2021). The heterogeneous pathways on HOXA11-AS/mRNA/miRNA pathways indicated a complicated yet fine network exist in keloids from within. Moreover, formidably high heterogeneity of HOXA11-AS-associated competing endogenous RNA (ceRNA) networks was also found in numerous malignant tumors (Reviewed by (Lu et al., 2018; Xue et al., 2018)). All these findings indicate HOXA11-AS functions both intensively and extensively with complicated molecular interaction and molecular cross-talk in keloids (reviewed by (Bayoumi et al., 2016; Lv et al., 2020)). In the present study, we computationally predicted an integrated interaction network for HOXA11-AS by its competing endogenous RNA network to seek intensively fine regulation of lncRNA–mRNA–miRNA network in benign tumor-like keloids using originally cultured keloid fibroblasts.

## Materials and Methods

### Obtaining Keloid Explants by Clinical Excision

Three anonymous patients affected with chronic keloids underwent keloid excision surgery in October 2019 in the Department of Plastic Surgery, China-Japan Union Hospital of Jilin University. All these keloids had been formed more than 1 year without any treatments and therapies. Keloid tissues were excised from each patient under aseptic conditions. The utilization of these removed samples from surgery was approved by the Medical Ethics Committee of Jilin University.

### Harvesting Primary Cultured Keloid-Derived Fibroblasts

All fresh samples were overtaken from central regions of respective excised keloid tissues (within half in length, width and depth). They were immediately punched into mini cube explants for further grinding into tiny pieces. Tiny explants were primarily cultured according to previous protocol with mild modification (Tucci-Viegas et al., 2010). All fresh keloid explants were washed with phosphate-buffered saline (PBS; Cultilab, SP, Brazil), penicillin (100 Ul/ml; Gibco, Carlsbad, CA, United States) and streptomycin (100 μm/ml; Gibco). Mini explants were incubated in Dulbecco’s modified Eagle’s medium (DMEM; Cultilab) for surface adherence. Consistent culture was conducted in DMEM adding 15% fetal bovine serum (FBS; Cultilab), penicillin (100 UI/ml; Gibco) and streptomycin (100 μg/ml; Gibco). Based on the previously studied platform, originally cultured keloid-derived fibroblasts were harvested and pooled for siRNA transfection and sequencing. FITC-labeled Ki-67 monoclonal antibody (Invitrogen, United States) was used to demonstrate the proliferation of the primary cultured keloid-derived fibroblasts from the central region. The staining of Ki-67 in cultured keloid fibroblasts was visualized by immunofluorescence microscopy.

### HOXA11-AS Knockdown in Keloid Fibroblasts *via* Small Interfering RNAs

Primary cultured keloid fibroblasts (Lowe passages:2–3) were used for siRNA interference using transfection approach. All siRNAs were designed and offered as commercial products (Sigma-Aldrich, United States). They were designed of the HOXA11-AS (ENSG00000240990). Sequences of three siRNAs were: GGUCCAACAGCCGAGCUUAdTdT/UAAGCUCGGCUGUUGGACCdTdT (Designed against HOXA11-AS, Sequence Start 856); CCAAGUCCGAGUUCCAUUUdTdT/AAAUGGAACUCGGACUUGGdTdT (Designed against HOXA11-AS, Sequence Start 923) and CGGCUAAUGCAAGAGGCCAdTdT/UGGCCUCUUGCAUUAGCCGdTdT (Designed against HOXA11-AS, Sequence Start 1099). Three 100 nM siRNA plasmids targeting HOXA11-AS were simultaneously transfected into two keloid fibroblasts (samples 1 and 2) respectively. Normal keloid fibroblasts were overtaken as a blank sample 0. To validate the siRNA transfection efficiency, quantitative polymerase chain reaction (qPCR) was conducted in keloid fibroblasts before and after HOXA11-AS knockdown (Forward primer:5′CGCTGACATCCGAGGAGAC3'; Reverse primer:5′ CTC​TTC​AAG​AAA​TGG​AAC​TCG3′). Significantly reduced expression of HOXAS-11 was shown in knockdown group (pooled sample 1 and 2) compared with normal group (sample 0) by three repeats, showing a successful RNA interference (Supplementary Figure S1). Simultaneously, plasmids harboring scrambled nonspecific RNAs were transinfected as a positive control (sample 3). A blank plasmid harboring no siRNA was transfected as a negative control (sample 4).

### Systematic Sequencing of lncRNAs, mRNAs, and miRNAs in Five Keloid Fibroblasts

The sequences of lncRNAs, mRNAs and miRNAs were respectively measured in five samples of keloid fibroblasts. Briefly, total RNAs or miRNAs of respective groups of cells were abstracted (Invitrogen Life Technologies), amplified and transcribed into fluorescent cRNA (Arraystar, Rockville, MD). The labeled cRNAs were hybridized and deeply sequenced (Aksomics, Shanghai). Identification of differentially expressed (DE) mRNAs, lncRNAs, and miRNAs in keloid fibroblasts with HOXA11-AS knockdown or control knockdown DE profiles of mRNAs, lncRNAs, or miRNAs were analyzed between HOXA11-AS-knockdown and normal keloid fibroblasts with a threshold fold change >1.5 and *p*-value ≤ 0.05. The DE profiles of the four studied samples compared with blank sample 0 were respectively screened by edgeR package and shown by heatmap. Keloid fibroblasts with HOXA11-AS knockdown (samples 1 and 2) and nonspecific knockdown (samples 3 and 4) were compared with normal keloid fibroblasts (sample 0). For DE profiles of keloid fibroblasts with HOXA11-AS knockdown, an intersection profile between samples 1 and 2 was used and shown by an intersection Venn diagram. For DE profiles in keloid fibroblasts with nonspecific knockdown, a union profile between samples 3 and 4 was used and shown by a union Venn diagram. To enhance the specificity of the resultant mRNAs and lncRNAs, the outcomes were identified as the HOX-A11-AS-knockdown profiles after removing overlapping profiles shown in the nonspecific controls (Shown by two intersection Venn diagrams).

### Construction of an Extended lncRNA–mRNA–Protein Network Based on lncRNA–mRNA Sense–Antisense Interaction in Keloid Fibroblasts

To screen potentially functioning mRNAs targeted by HOXA11-AS using an integrated analysis, an interactive lncRNA–mRNA network was initially constructed based on sequence matching as well as loci proximity (Tsai et al., 2010; Ulitsky et al., 2011; Derrien et al., 2012) using human lncRNA–mRNA bioinformatic prediction in the HOXA11-AS-knockdown group or in the control group. Next, an extended LMP interaction network was further constructed by integration into the human protein–protein interaction (PPI) network for screening dominant functional mRNAs. A human PPI database was abstracted using Cytoscape 3.9.0 (http://manual.cytoscape.org/en/stable/). Neighboring proteins proximate to DE mRNAs 100 within the lncRNA–mRNA interaction network were overtaken to construct a first-neighbor network. With the aid of the first-neighbor network, the DE mRNAs interacting with DE lncRNAs that undergo crosstalk with HOXA11-AS were identified in keloid fibroblasts. Using the same protocol, first-neighbor proteins linked to the lncRNA–mRNA network were also integrated into the LMP interaction network in control fibroblasts.

### Screening Dominant Functional mRNAs in Keloid Fibroblasts With HOXA11-AS Knockdown Using the PageRank Algorithm

Based on the principle of random walk, the PR algorithm was used to score and rank the potential dominance of the functioning mRNAs in the integrated LMP network in HOXA11-AS-knockdown keloid fibroblasts. This approach was employed so that each node could be exclusively ranked by its PR value within an integrated network of which cumulative PR values of all composing nodes equaled 1. Therefore, the PR value of a specific mRNA was used to represent for its functional dominance in the integrated LMP network. Next, mRNAs with below average PR values were removed as non-dominantly functioning genes. To enhance the specificity, the mRNAs that overlapped with those in the control group were removed. Gene Ontology (GO; http://geneontology.org/) analysis and KEGG analysis (https://www.genome.jp/kegg/) were performed to determine the enrichment of the screened dominant functional genes in functional pathways. Unenriched genes were removed from the outcome profile. GO and KEGG analysis was not performed for control samples.

### Validation of Dominant Functional Genes by Quantitative Polymerase Chain Reaction and Western Blotting in Keloid Fibroblasts With or Without HOXA11-AS

For the genes screened by PR value, qPCR was conducted in both normal and HOXA11-AS-knockdown keloid fibroblasts according to the manufacturer’s manual (Aksomics, China). The primer sequences of screened genes are shown in Supplementary Table S1. All qPCRs were conducted three times. For mRNAs that underwent PCR validation, western blotting was further conducted in normal and in HOXA11-AS-knockdown keloid fibroblasts. In this study, the monoclonal antibodies used were anti-VTP (Abcam, ab113065, United States, diluted 1:300), anti-SNED1 (EnoGene, E021040, United States, diluted 1:4,000), and anti-NIPAL3 (Abcam, ab32419, diluted 1:1,000).

### Constructing an Endogenous Competing Network of HOXA11-AS–DE miRNA(s)–Validated mRNA(s) in Keloid Fibroblasts

As numerous studies showed that miRNAs are substantially involved in regulation *via* competing endogenous RNA (ceRNA) networks, we attempted to construct a ceRNA network between HOXA11-AS and validated mRNAs in keloid fibroblasts with HOXA11-AS knockdown. An expression-based competition was screened as follows: downregulated lncRNA of HOXA11-AS–upregulated miRNA(s) among DE miRNA profile–downregulated validated mRNA(s). Simultaneously, DE miRNAs were screened by sense–antisense sequence matching according to a human mRNA–miRNA database using the TargetScan 5.0 online tool (http://www.targetscan.org/vert_80/). Consequently, dual validated miRNAs that underwent intersection between two resultant profiles were identified as competing endogenous miRNAs. Finally, overlapping miRNAs from the control group were removed to enhance the specificity.

## Results

### Primary Cultured Keloid Fibroblasts From Central Regions of Chronic Keloid Tissues

**FIGURE 1:**
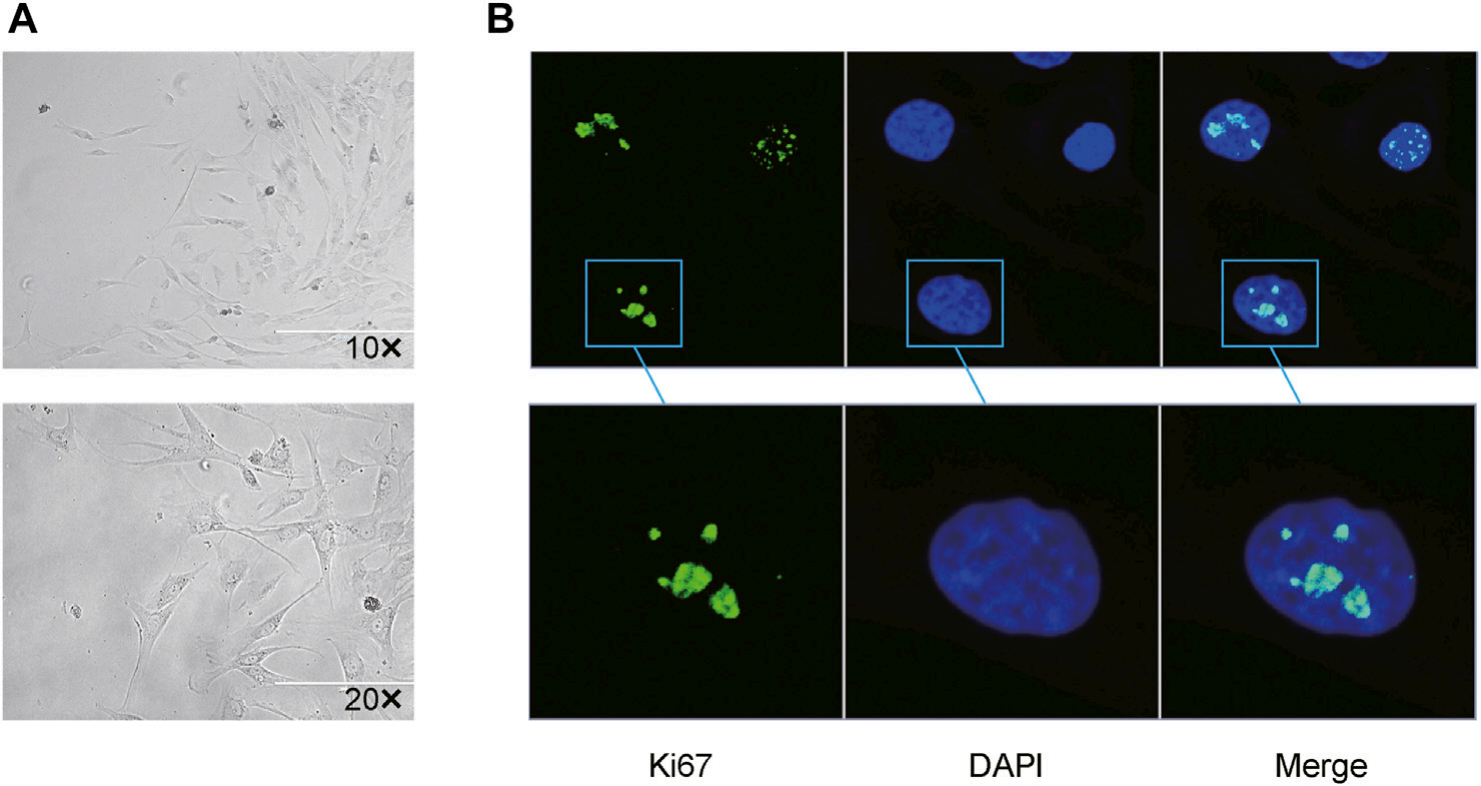
Successful primary culture of keloid fibroblasts from the central region of the chronic keloids **(A)** Keloid fibroblasts could be observed via optical microscopy after 15 days of primary culture from 4 mini explants in the central regions (4/22) of the excised keloid tissues (Bar: 200 µm). **(B)** Using Ki-67 as a marker of cell proliferation, low-to-moderate proliferation (green) was observed in 2 passaged keloid fibroblasts from central regions of keloid tissue, with DAPI-stained cell nuclei (blue) co-expression.

All mini keloid explants were overtaken from central regions of the excised keloid tissues to reduce the proportion of normal fibroblasts. Resultantly, merely four mini-keloid explants (4/22) could be successfully cultured. All four keloid explants were found from the edges of the central regions of excised chronic keloid tissues. Using optical microscopy, the keloid fibroblasts from central regions of keloid tissues could be visualized after 10 days of culture (Figure 1A). Using the proliferative marker of Ki-67 antibody, mild-to-moderate proliferation in the keloid fibroblasts was observed *via* confocal microscope (Figure 1B).

**FIGURE 2:**
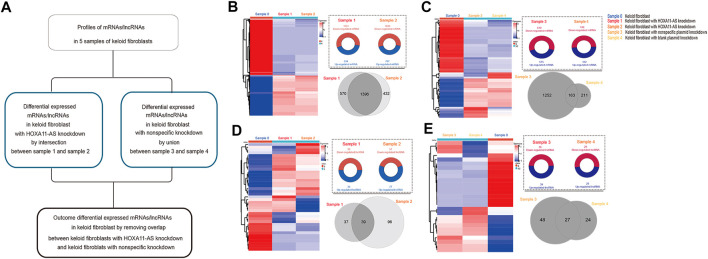
Messenger RNA (mRNA) and long noncoding RNA (lncRNA) expression heatmap and differentially expressed (DE) profiles in keloid fibroblasts with HOXA11-AS knockdown stepwise flowchart for outcome differentially expressed (DE) mRNAs/lncRNAs in keloid fibroblasts with HOXA11-AS-knockdown with removal of nonspecific overlap **(A)**. Expression heatmap of DE mRNAs in keloid fibroblasts with HOXA11-AS knockdown (**(B)**, left). Upregulated and down-regulated profiles between samples 0 and 1 as well as between samples 0 and 2 were intersected for the DE mRNAs in with HOXA11-AS knockdown (**(B)**, right). Expression heatmap of DE mRNAs in keloid fibroblasts with nonspecific control knockdown (**(C)**, left). Both upregulated and down-regulated profiles between samples 0 and 1 as well as between samples 0 and 2 were intersected for the DE mRNAs with nonspecific control knockdown (**(C)**, right). Expression heatmap of DE lncRNAs in keloid fibroblasts with HOXA11-AS knockdown (**(D)**, left). Both upregulated and down-regulated profiles between samples 0 and 3 as well as between samples 0 and 4 were united for DE lncRNAs in with HOXA11-AS knockdown (**(D)**, right). Expression heatmap of DE mRNAs in keloid fibroblasts with nonspecific control knockdown (**(E)**, left). Upregulated and down-regulated profiles between samples 0 and 3 as well as between samples 0 and 4 were united for the DE lncRNAs with nonspecific control knockdown (**(E)**, right).

Based on these low-passaged keloid-derived fibroblasts, the differential profile targeting HOXA11-AS were compared. HOXA11-AS knockdown altered the mRNA and lncRNA profiles in keloid fibroblasts DE lncRNAs were identified in keloid fibroblasts with HOXA11-AS knockdown by intersection profile between samples 1 and 2. For the nonspecific knockdown profile, DE lncRNAs were identified by a union profile that was constructed for samples 3 and 4 to enhance the specificity of the outcome. The final DE lncRNAs were identified by removing overlapping lncRNAs between the HOXA11-AS-knockdown and control groups. The flowchart was shown in Figure 2A. Using heat map expression and Venn diagrams, DE mRNA and lncRNA profiles in keloid fibroblasts with HOXA11-AS knockdown as well as with nonspecific control knockdown were visualized respectively (Figures 2B–E). Intersection of samples 1 and 2 produced a total of 1,396 DE mRNAs in keloid fibroblasts with HOXA11-AS knockdown. The union of nonspecific and blank knockdown also produced 1,626 DE genes in keloid fibroblasts, and was collectively referred to as the nonspecific knockdown. Only a few DE lncRNAs were shown by knockdown of the lncRNA HOXA11-AS, indicating crosstalk between lncRNAs does exist. All DE mRNAs and significant DE mRNAs are shown in Supplementary Datasets 1S and 2S, respectively. All DE lncRNAs and significant DE lncRNAs are shown in Supplementary Datasets 3S and 4S, respectively.

### Integrating HOXA11-AS-Associated lncRNA–mRNA Interactions by Constructing an Extended Network Using DE lncRNAs and mRNAs to Screen Dominant Functional Genes by PR Algorithm Targeting HOXA11-AS in Keloid Fibroblasts

**FIGURE 3:**
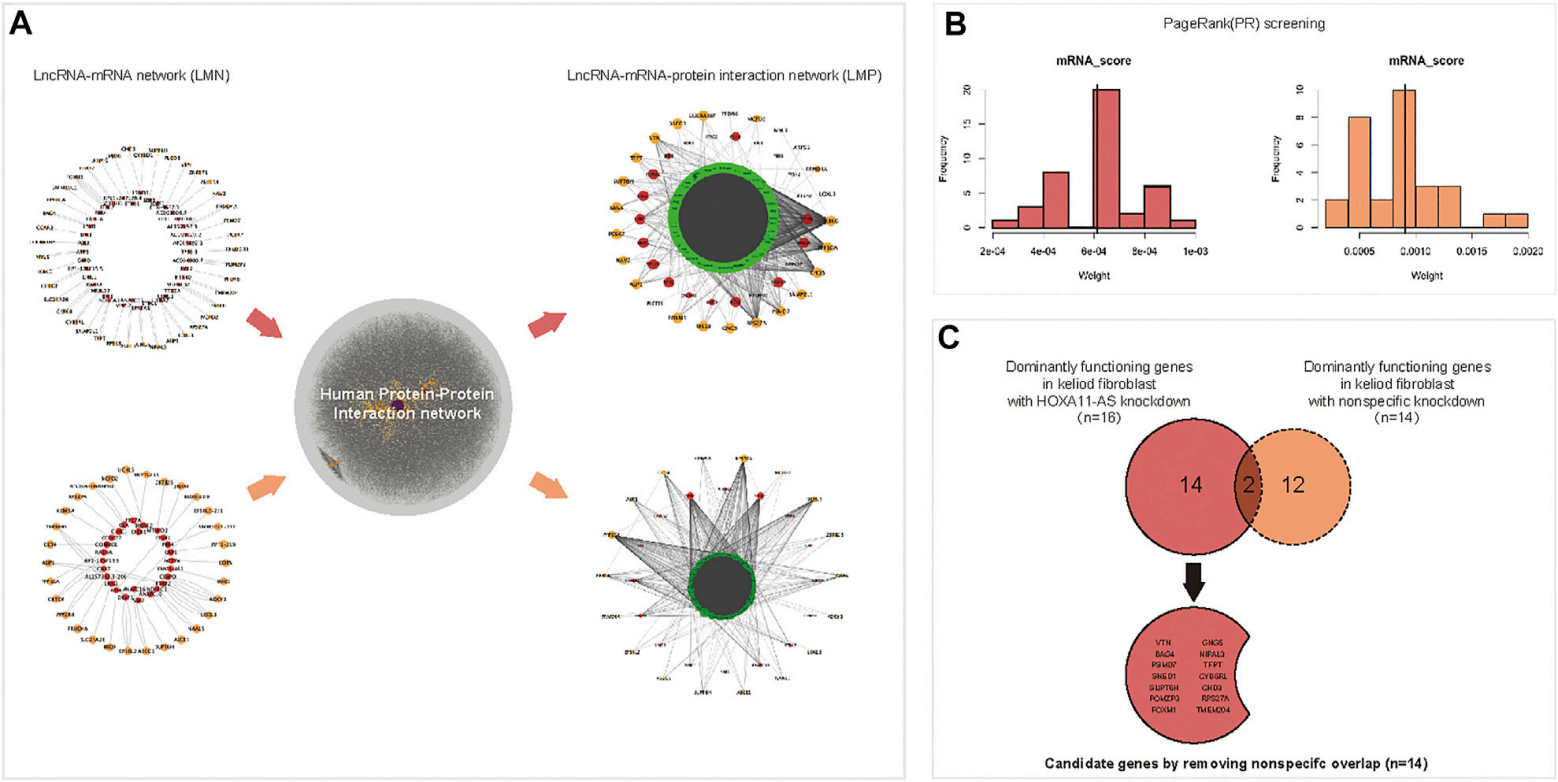
Predicting dominant functional mRNAs in the HOXA11-AS regulatory network of keloid fibroblasts using interaction network construction and PageRank (PR) scoring. A lncRNA–mRNA interacted network was constructed using differentially expressed (DE) lncRNAs and DE mRNAs in keloid fibroblasts with HOXA11-AS knockdown based on cite- and sequence-matched prediction. A total of 39 lncRNAs (red nodes) corresponding to 63 mRNAs (orange nodes) were predicted by knocking downHOXA11-AS in keloid fibroblasts (**(A)**, upper, left). Using the same protocol, a lncRNA–mRNA network was constructed with 28 lncRNAs (red nodes) corresponding to 30 mRNAs (orange nodes) in keloid fibroblasts with nonspecific knockdown (**(A)**, lower, left). An integrated lncRNA–mRNA–protein network using first-neighbor genes (green band) linked to 39 lncRNAs (red nodes) and 63 mRNAs (orange nodes) were identified using DE mRNA profiles in keloid fibroblasts with HOXA11-AS-knockdown with the aid of the human protein–protein–interaction annotated network (**(A)**, upper, right). Using the same protocol, the integrated lncRNA–mRNA–protein network was constructed in keloid fibroblasts with nonspecific knockdown (**(A)**, lower, right). A total of 23 mRNAs were predicted to be dominantly functioning mRNAs screened by the PR algorithm in keloid fibroblasts with HOXA11-AS knockdown (**(B)**, left). Using the same protocol, 14 mRNAs were predicted in keloid fibroblasts with nonspecific knockdown (**(B)**, right). Among the 23 mRNAs, 16 mRNAs that were enriched in biological pathways in keloid fibroblasts were predicted to be involved in the HOXA11-AS pathway, two were also predicted to be involved in nonspecific knockdown and were removed as “noise.” Consequently, 14 candidate mRNAs were determined to be regulated by HOXA11-AS in keloid fibroblasts **(C)**.

The DE lncRNAs and mRNAs which were predicted as intronic antisense or natural antisense interrelation based on genome cite were screened using bioinformatics analysis (Supplementary Dataset 5S). The resultant lncRNA-mRNA interaction network, which was characterized with both expression- and cite-matching, included 39 lncRNA nodes and 63 mRNA nodes (Figure 3A, upper left). To screen dominant functional genes, an extended network that was re-constructed using the human PPI network could reveal more complicated internal crosstalk (The online data extracted for Human PPI network were shown in Supplementary Dataset 6S. The network of Human PPI was shown in Supplementary Material S1). Based on proximity-based “neighbor” proteins, an integrated network between 39 interacting lncRNAs and 63 interacting mRNAs was re-constructed, which produced a first neighbor-associated LMP network (Figure 3A, upper right). This LMP network contained 929 nodes and 6,572 edges, and integrated 39 interacting DE lncRNAs and 63 interacting mRNAs. Using the same protocol, 28 lncRNAs interacting with 30 mRNAs were screened in the control group (Figure 3A, lower left). An integrated LMP network based on the human 180 PPI network was also constructed for the control group (Figure 3A, lower right).

Using PageRank algorithm, 23 mRNAs harboring above average PR scores remained as dominant functional genes targeting HOXA11-AS in keloid fibroblasts. Among them, 16 genes enriched into biological pathways by online GO were remained for HOXA11-AS-knockdown keloid fibroblasts (Figure 3B, left). A total of 14 mRNAs with sufficiently high PR scores were screened in the nonspecific group (Figure 3B, right). The 16 enriched mRNAs from the HOXA11-AS group intersected the 14 screened mRNAs from the nonspecific group; the two overlapping mRNAs were removed. The remaining 14 mRNAs from the HOXA11-AS group were determined to have dominant functions in the HOX11A-AS-asssociated regulatory network (Figure 3C).

### Validation of 14 mRNAs Targeting HOXA11-AS in Keloid Fibroblasts Using qPCR and Western Blotting

**FIGURE 4:**
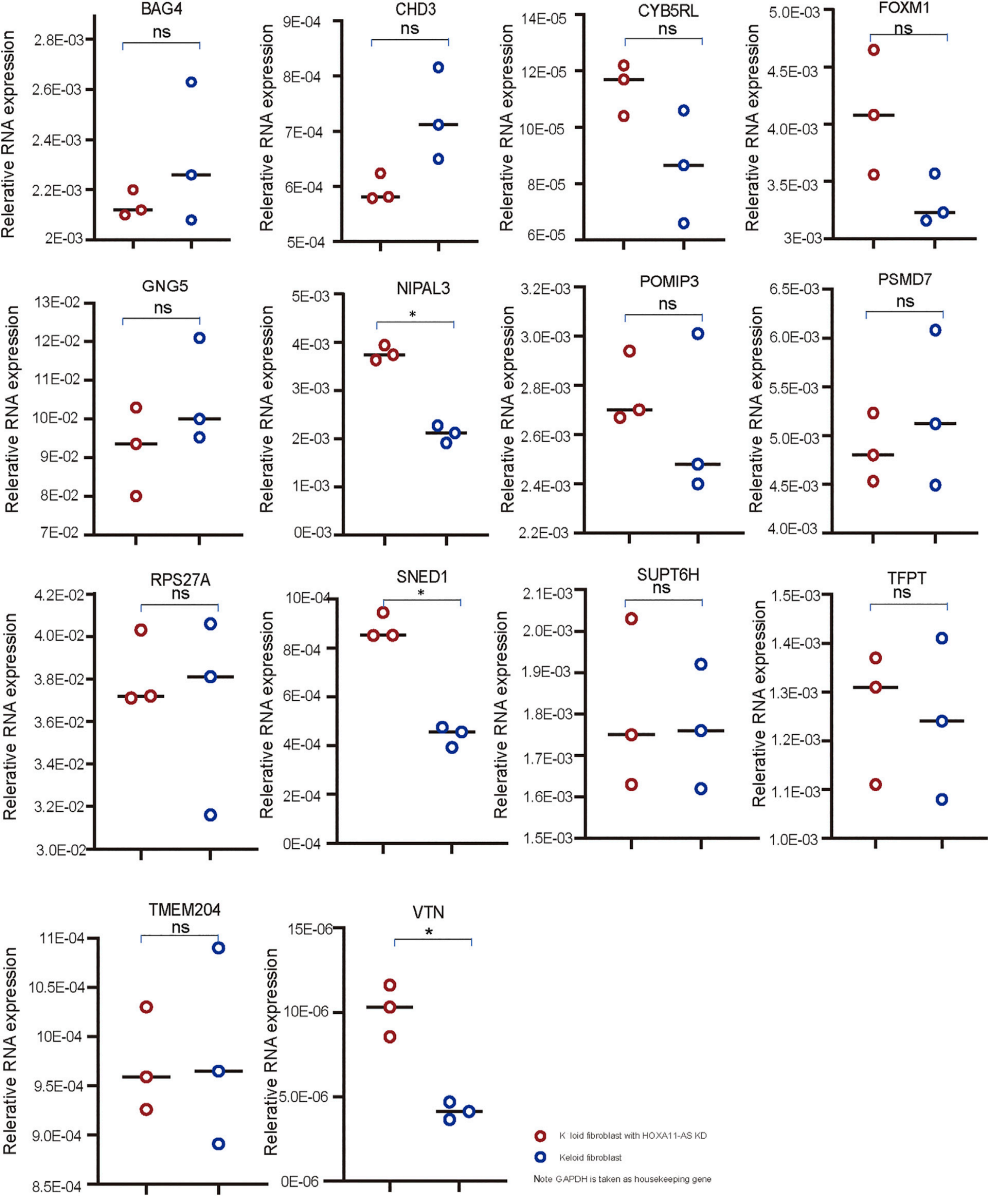
qPCR validation in keloid fibroblasts with HOXA11-AS knockdown Three mRNAs, VTN, SNED1, and NIPAL3, were found to have significantly altered expression in keloid fibroblasts with HOXA11-AS knockdown based on qPCR validation (* *p* < 0.05: ns, non significant).

Using qPCR, three genes (SNED1, NIPAL3, and VTN) among 14 candidate genes were shown to be significantly changed by HOXA11-AS-knockdown keloid fibroblasts compared with normal keloid fibroblasts (Figure 4). Therefore, these genes were considered to be dominantly functioning in the HOXA11-AS1-involved network. Using function enrichment analysis, these genes were enriched in three pathways (R-HAS-5689603: UCH proteinases; M3008: NABA ECM glycoprotein; GO 0071363: cellular response to growth factor stimulus). The results indicated the interactive network targeting HOXA11-AS extensively and intensively function to endogenous genes at cellular level. However, no detectable expression of SNED1, NIPAL3, and VTN was shown in either normal or HOXA11-AS-knockdown keloid fibroblasts using western blotting (data not shown). We attributed the negative outcome to the very low-level expression of endogenous genes that were barely detected using western blotting, which has much lower sensitivity than qPCR.

### Screening Competing Endogenous miRNAs by Construction of the HOXA11-AS-Regulatory Interaction Network in Keloid Fibroblasts

**FIGURE 5:**
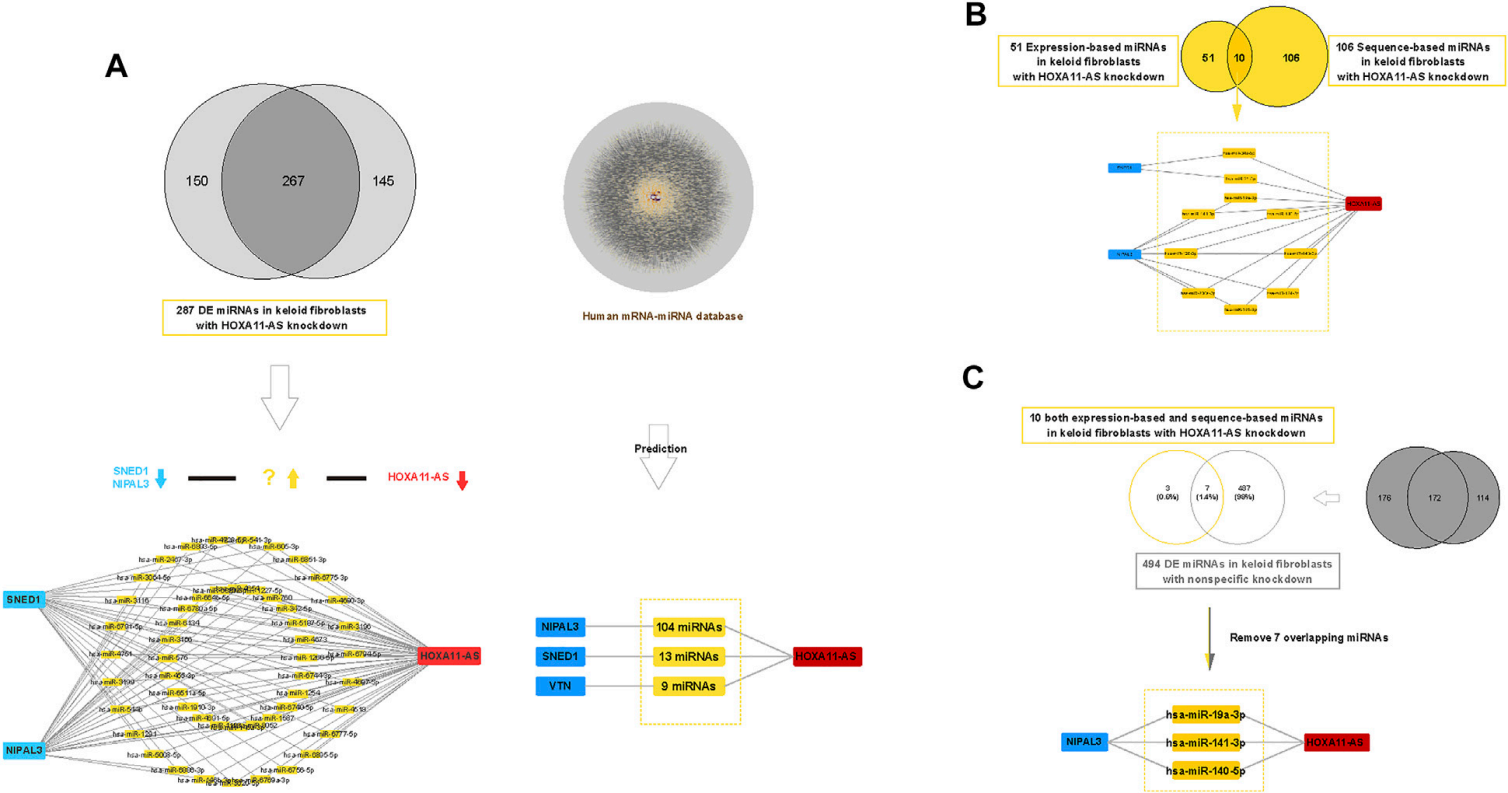
Construction of a competing endogenous RNA (ceRNA) network of HOXA11-AS in keloid fibroblasts. Expression-based down regulated (HOXA11-ASknockdown)–up regulated (DE miRNAs)–down regulated (SNED1 and NIPAL3) ceRNA network constructed in keloid fibroblasts showing 51 upregulated miRNAs (**(A)**, left). Expression-based Sequence-based prediction screened 106 unique competing endogenous miRNAs against HOXA11-AS based on two human predictive databases (lncRNA–miRNA and miRNA–mRNA) (**(A)**, right). Based on dual prediction, there were 10 screened candidates sponging miRNAs in the HOXA11-AS–NIPAL3 regulatory pathway **(B)**. After removing seven overlapping miRNAs that were shown among 494 nonspecific miRNAs, three upregulated miRNAs (hsa-miR-19a-3p, hsa-miR-141-3p, and hsa-miR-140-5p) were identified as outcome sponging miRNAs in the HOXA11-AS–NIPAL3 regulatory pathway in keloid fibroblasts **(C)**.

We revealed three validated genes involved in the HOXA11-AS-associated network in keloid fibroblasts. Then, we screened potential miRNAs that act as ceRNAs in the network using both sequence-matched and expression-matched validation. Based on DE miRNA profiles in keloid fibroblasts with HOXA11-AS knockdown or control knockdown (Supplementary Datasets 7S and 8S), we screened potential competing endogenous miRNAs step by step (Figure 5). First, we screened expression-matched molecular pairs by downregulated (HOXA11-AS knockdown)–upregulated (DE miRNAs)–downregulated (NIPAL3 and SNED1) regulation. We predicted 51 upregulated DE miRNAs (Figure 5A, left). Subsequently, we predicted sequence-matched miRNAs that interacted with NIPAL3 (104 miRNAs), SNED1 (13 miRNAs), and VTN (9 miRNAs) among the DE miRNA profiles; this HOXA11-AS-dependent mRNA-miRNA screen was determined by referencing the human mRNA–miRNA competing endogenous RNA database (Predicted human miRNA-mRNA interaction dataset was shown in Supplementary Dataset 9S. Predicted human miRNA-mRNA interaction network was shown in Supplementary Material S2). By removing 20 overlapping miRNAs, we identified 106 miRNAs (Figure 5A, right). Third, we intersected sequence-based miRNAs with expression-based miRNAs to address the 10 upregulated miRNAs (Figure 5B). Fourth, to remove the nonspecific control, we intersected 10 miRNAs with 495 upregulated DE miRNAs in the control group (List of 495 upregulated DE miRNAs in control samples were shown in Supplementary Dataset 10S), which revealed three miRNAs (hsa-miR-19a-3p, hsa-miR-141-3p, and hsa-miR-140-5p) by removing the seven overlapping miRNAs (Figure 5C).

## Discussion

**FIGURE 6:**
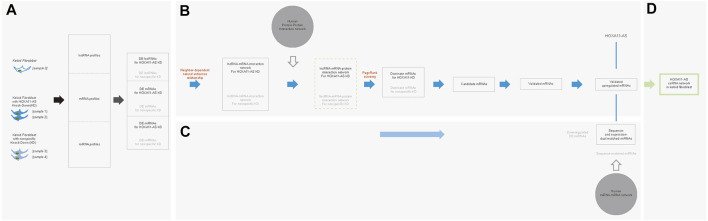
Stepwise bioinformatic analysis of the HOXA11-AS-interactive molecular network in keloid fibroblasts. Five keloid fibroblast samples were sequenced and compared to create three differentially expressed (DE) profiles of lncRNAs, mRNAs, and miRNAs betweenHOXA11-AS-knockdown and normal samples as well as between nonspecific control knockdown and normal samples. **(A)**. Based on DE lncRNA and mRNA profiles, an interactive network was initially constructed based natural/intronic antisense neighbor-dependent relationship. Based on the network, a lncRNA-mRNA-protein network was extended mediated by the human protein–protein interaction network. Within the integrated network, screened DE mRNAs were ranked by PageRank score to identify dominant functional mRNAs. Candidate mRNAs were *in vitro* validated by qPCR and western blotting. All overlapping molecules between the study and nonspecific control groups were removed for each step **(B)**. Validated mRNAs were considered target genes and screened for competing endogenous mRNAs based on DE miRNAs. Simultaneously, these competing endogenous miRNAs underwent sequence-based validation. **(C)**. By the stepwise bioinformatic screening, an integrated molecule network of HOXA11-AS–three miRNAs–NIPAL3 interactions was shown in keloid fibroblast **(D)**.

Our team had compared the lncRNA profiling between keloids and peripheral normal skin and revealed a significant increase of HOXA11-AS in keloids (Sun et al., 2017). In the present study, we used mechanically separated keloid-derived fibroblasts from the central regions of excised keloids to mimic the original molecular environments harboring both scar-like and cancer-like characters. By monitoring dynamic changes of proliferative marker (Ki-67), we found that keloid-derived fibroblasts from central regions display slow yet relative homogenous trajectories than those from peripheral regions (Supplementary Figure 2S). In the present study, we successfully obtained low-passaged keloid fibroblasts from central regions of keloids. We supposed these keloid-derived fibroblasts which were mixed with low proportion of normal fibroblasts highly mimicked the molecule environment of well-transformed keloid fibroblasts. We harvested these keloid fibroblasts for the HOXA11-AS knockdown, nonspecific knockdown, and blank knockdown to predict an integrated network targeting HOXA11-AS. It has been well acknowledged that an intertwined molecular network cooperatively functioned to a molecule of interest (here is HOXA11-AS) by extensive crosstalk, interactive cooperation, and competitive co-regulation (Sasaki et al., 2007; Morris and Mattick, 2014; Bayoumi et al., 2016; Yamamura et al., 2018; Li et al., 2019). Therefore, we attempted to construct such an integrated network for HOXA11-AS in keloid fibroblasts with respect to the extensive crosstalk between lncRNAs, mRNAs, and miRNAs using different combinations of computational methods (Figure 6; All codes in the present study were shown in Supplementary Material S1).

We transfected three designed siRNAs from different sequence starts to “knockdown” HOXA11-AS in two keloid fibroblast samples to construct the cellular model. Additionally, we obtained the intersection of the profiles between these two samples to enhance specificity. We simultaneously constructed two control samples to reduce the nonspecific “noise”. We then removed the combined “noises” of two control samples to further enhance the specificity When we identified DE lncRNA and mRNA profiles with high specificity, we next identified lncRNA-mRNA interrelation by both natural/intronic antisense relationship and loci proximity using bioinformatics prediction. We obtained 63 DE mRNAs that closely interacted with 36 DE lncRNAs that were associated with HOXA11-AS in keloid fibroblasts. Using the well-validated human PPI network, we extended the DE lncRNA–mRNA interactive association with respect to extensive crosstalk between molecules in cell. The extended interaction network contained 929 nodes and 6,572 edges, which included 22 DE lncRNAs and 25 DE mRNAs associated with HOXA11-AS. Presented as an integrated network, we computationally ranked these molecules by putative dominance using the PR algorithm. After we identified 25 DE mRNAs with above-average PR scores, we considered these genes to have putatively dominant functions. Among these 25 mRNAs, merely 16 were enriched in biological pathways. After removing two overlapping mRNAs in the control, we identified 14 mRNAs with putatively dominant functions involved in the integrated HOXA11-AS network in keloid fibroblasts. Intriguingly, the 14 genes were enriched in three pathways for transcriptional regulation and signaling transduction, which is globally consistent with the known pathways of endogenous homeobox (HOX) genes (Shen et al., 2001; Abate-Shen, 2002).

We then conducted *in vitro* validation using both qPCR and western blotting. We obtained three PCR-validated genes (NIPAL3, SNED1, and VNT) without any detectable proteins by western blotting, even in normal keloid fibroblasts without HOXA11-AS knockdown. We interpreted the negative blotting as being related to the very low expression of these endogenous genes in keloid fibroblasts.

To further screen potentially competing endogenous miRNAs in the HOXA11-AS-associated integrated regulatory network, we predicted a ceRNA network using HOXA11-AS and three validated genes (NIPAL3, SNED1, and VNT). For sequence-based validation, we screened 108 DE miRNA antisense sequences against the three genes based on the predicted human miRNA–mRNA interaction database. For expression-based validation, we screened competing endogenous miRNAs in the downregulated (HOXA11-AS knockdown)–upregulated (DE miRNAs)–downregulated (PCR-validated NIPAL3 and SNED1) network. By intersection of sequence- and expression-matched miRNAs, we identified 10 miRNAs. After removing seven overlapping miRNAs from the nonspecific knockdown group, we identified the outcome ceRNA network of the HOXA11-AS (hsa-miR-19a-3p, hsa-miR-141-3p, and hsa-miR 140-5p)–NIPAL3 regulatory pattern in keloid fibroblasts.

The three screened miRNAs, hsa-miR-19a-3p, hsa-miR-141-3p, and hsa-miR-140-5p, were previously shown to be aberrantly expressed in various malignant tumors. However, they also show opposite regulation for the one specific function. For example, epithelial mesenchymal transition (EMT) is an essential mechanism of keloid formation; in different tumors, hsa-miR-19a-3p promotes EMT, whereas hsa-miR-141-3p and hsa-miR-140-5p inhibit EMT (Hahn et al., 2016; Kuwahara et al., 2016; Yang et al., 2018). As HOXA11-AS was demonstrated to promote cellular proliferation and tumorigenesis, we propose that real-time regulation of HOXA11-AS-involved integrated network in keloid fibroblast might be determined by the cooperatively expressed miRNAs.

In this study, the HOXA11-AS-associated molecular network was identified by stepwise bioinformatic analysis. Such computational screening revealed an integrated complexity of interrelationship between miRNAs, genes, and HOXA11-AS in keloid fibroblasts. The present molecular network has high specificity with much reduced subjective bias. Additionally, among DE molecules associated with HOXA11-AS knockdown, we predicted DE lncRNAs and mRNAs interaction by site- and sequence-dual interrelation. Notably, the lncRNA profile was slightly impacted by HOXA11-AS knockdown, which was due to lncRNA–lncRNA crosstalk, either by co-miRNAs or by co-mRNAs(S Yamamura et al., 2018; Li et al., 2019). Therefore, our analysis intensively revealed the integrative interaction of the lncRNA–miRNA–mRNA network from within. When we identified an extended integrated network using the human PPI network, we screened dominant functional genes using PR algorithm scoring of each molecule within the network. All 14 genes we screened were associated with an endogenous gene HOX family, which indicated that the present molecular network had good specificity. Moreover, three DE profiles were identified by removing overlap with the nonspecific control knockdown group, which further reduced “noise” during high-throughput data analysis.

Our study has three limitations. First, merely regulatory pattern *via* competing endogenous RNAs was focused on. Second, the validation sample size was small, which reduced the sensitivity of the results. Third, western blotting could not detect protein-level alterations, which indicated that the complicated endogenous network was not well reflected by clinical phenotypes. A stable overexpression of HOXA11-AS in keloid fibroblast is required to further validate the present interaction network.

## Conclusion

Using integrated bioinformatic analysis and *in vitro* validation, a HOXA11-AS-associated interactive network was shown in keloid fibroblasts.

## Data Availability

The datasets presented in this study can be found in online repositories. The name of the repository and accession number can be found below: National Center for Biotechnology Information (NCBI) Gene Expression Omnibus (GEO), https://www.ncbi.nlm.nih.gov/geo/, GSE191267.

## Supplementary Material

Supplementary Figure S1 Significantly downregulated HOXA11-AS in keloid fibroblasts with HOXA11-AS knockdown comparing to keloid fibroblasts without HOXA11-AS knockdown via qPCR.

Supplementary Figure S2 Comparison of dynamic expression of Ki-67 in three passages of keloid fibroblasts between central regions and peripheral regions within excised keloid samples.

## Abbreviations

lncRNA: long noncoding RNA; mRNA: messenger RNA; miRNA: microRNA; hsa-miR: human miRNA- MicroRNA; DE: differentially expressed; qPCR: quantitative polymerase chain reaction; PR: PageRank; ceRNA: competing endogenous RNA.
