# Supplementary figures and images for: Construction of a HOXA11-AS-Interacted Network in Keloid Fibroblasts Using Integrated Bioinformatic Analysis and in Vitro Validation
Source: Front Genet. 2022 Mar 31;13:844198. doi: 10.3389/fgene.2022.844198 (PMC9010035; doi:10.3389/fgene.2022.844198)

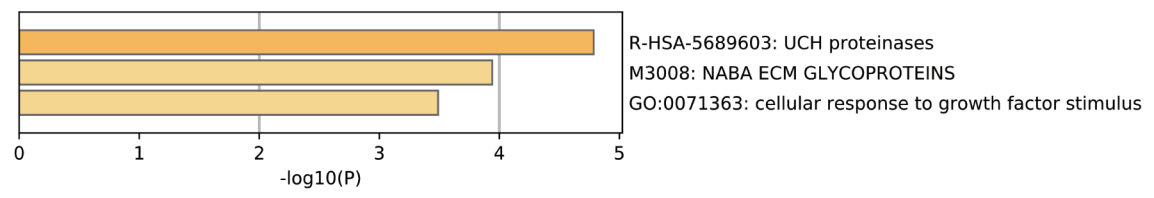

Supplement: Supplementary file 2 [file DataSheet4.PDF]

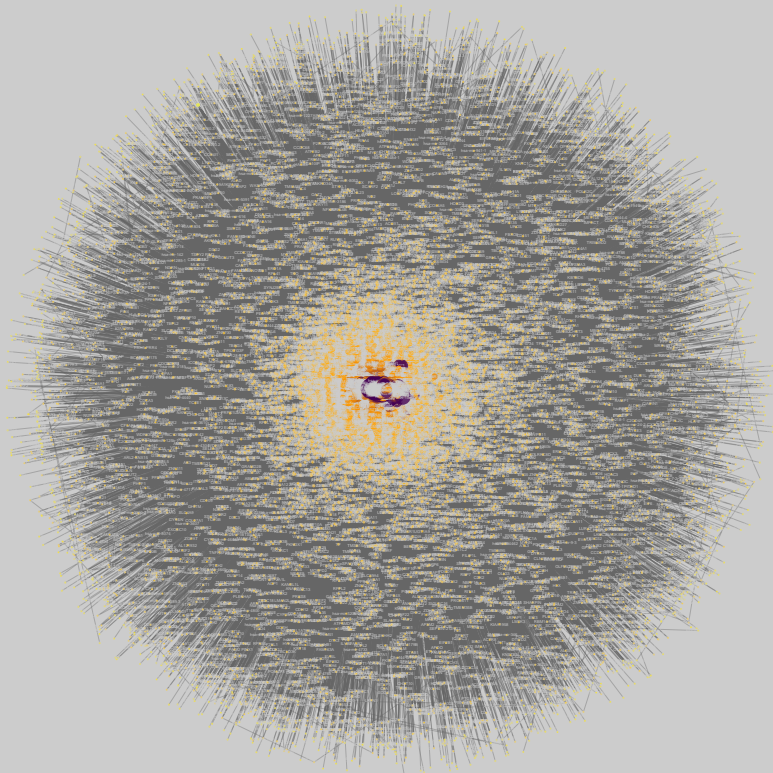

Supplement: Supplementary file 3 [file DataSheet6.PDF]

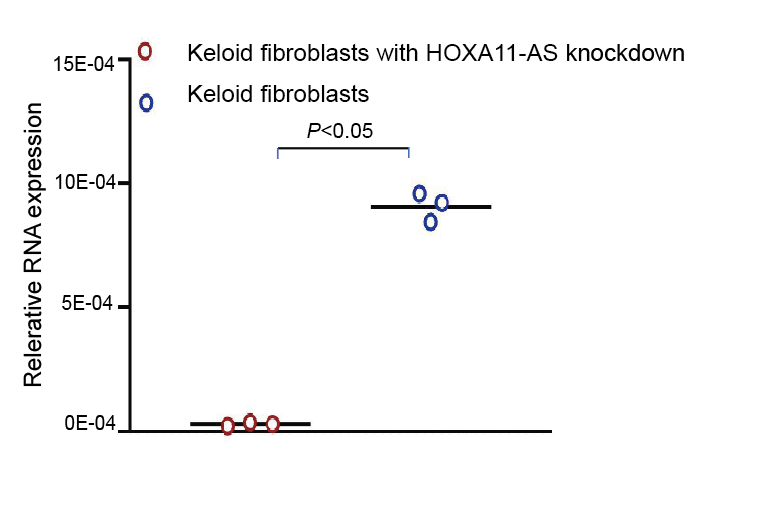

Supplement: Supplementary file 8 [file Image1.TIF]

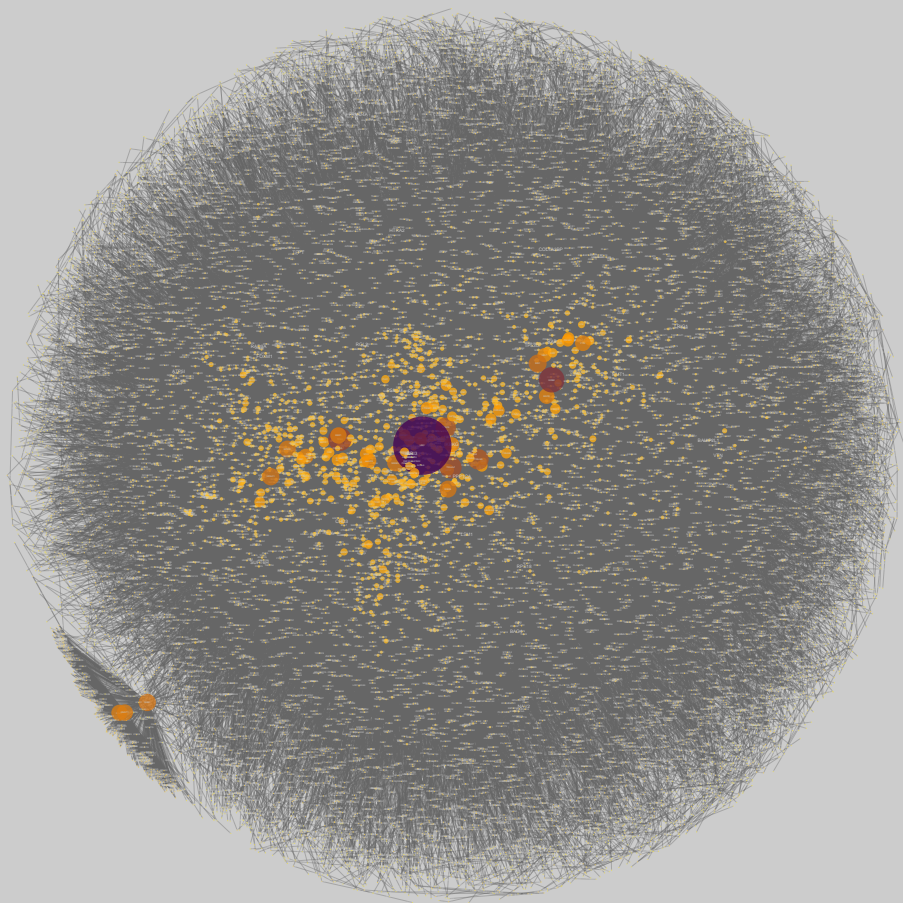

Supplement: Supplementary file 12 [file DataSheet5.PDF]
