# Supplementary material for: Construction of a HOXA11-AS-Interacted Network in Keloid Fibroblasts Using Integrated Bioinformatic Analysis and in Vitro Validation
Source: Front Genet. 2022 Mar 31;13:844198. doi: 10.3389/fgene.2022.844198 (PMC9010035; doi:10.3389/fgene.2022.844198)
Supplement: Supplementary file 6 [file Table8.DOCX]

Supplementary Table S1: Primer sequences of 14 candidate genes for PCR validation

| VTN | F:5'GGAGCGGGTCTACTTCTTCA3’, R:5’ ATGAACTGGGGCTGTCTGGT3’; |
| --- | --- |
| BAG4: | F:5' TATGGAATGGGTGGCCGTTAT3’, R:5’ CCCGAGGATTCGTACTGATGG 3’; |
| PSMD7 | F:5'GGTTGGAAACCAGAAGCGTG3’, R:5’ GTAGTTTAGGGCCTGTGTGGT3’; |
| SNED1: | F:5'AAAGCACAAGCCTCAAGAAGAC3’, R:5’ TTGGTGGAGTGTAACAAGAACG3’; |
| SUPT6H: | F:5' AAATCCGAGCCACTGACCTG3’, R:5’ GGTTGGTGTGGCAAAAGCAT3’; |
| POMZP3 | F:5' ACTGTACTGAGTGCCCTCAAAGA3’, R:5’ AAAGCCCACTGCTCTACTTCAT3’; |
| FOXM1 | F:5' AGCAGGCTGCACTATCAACA 3’, R:5’ TCAACCTTAACCTGTCGCTG 3’; |
| TMEM204 | F:5' GCCCTGGTCTCACTCATCCT 3’, R:5’ GCGCATCATGTCGAACTGC 3’; |
| GNG5 | F:5' CAAATCCCTTCAGACCCCAGA 3’, R:5’ TTGTATGCTGCTGCCAGTGTAT 3’; |
| NIPAL3 | F:5' CATTCGCACCCAACAGTCAC 3’, R:5’ AAAAGGAAAGGCCAGCTCAC 3’; |
| TFPT | F:5' GCAGAGGATAACTCGGAGGC 3’, R:5’ CAGCACAATGGTGAACTGGC 3’; |
| CYB5RL | F:5' GACTGGGAGGAACCTGGAAA 3’; R:5’ GCCAGGCTCTATGAGACCAC 3’; |
| CHD3 | F:5' TGCTGCAACCATCCATACCT 3’, R:5’ CGCTCATACTTGTAGCCTTCATAG 3’; |
| RPS27A | F:5' AGAAAGAAGGTTAAGCTGGCTGT 3’, R:5’ CTCTCGACGAAGGCGACTAAT 3’. |
