# Supplementary material for: Construction of a HOXA11-AS-Interacted Network in Keloid Fibroblasts Using Integrated Bioinformatic Analysis and in Vitro Validation
Source: Front Genet. 2022 Mar 31;13:844198. doi: 10.3389/fgene.2022.844198 (PMC9010035; doi:10.3389/fgene.2022.844198)
Supplement: Supplementary file 9 [file Table9.DOCX]

Supplementary Word 1S Key codes for constructing HOXA11-AS-associated integrated network in keloid fibroblasts

**Part One DE mRNA profiles (R)**

library(pheatmap)

setwd("/Users/guyue/Desktop/lnc_mir/ceRNA/diff/")

expres<-read.table("expression.txt",sep="\t",header=T,row.names=1,stringsAsFactors=F,comment.char = "")

KR_list<-read.table("K_R.txt",sep="\t",header=F)

diff_exp<-expres[expres[,1]%in%as.character(KR_list[,1]),c(1,5,7,8)]

write.table(diff_exp,"diff_exp_KR.txt",quote=F,sep="\t",row.names=T,col.names=T)

heat_data<-as.matrix(diff_exp[,c(2:4)])

heat_data=apply(heat_data,2,as.numeric)

heat_data=heat_data[-127,]

heat_data=scale(heat_data,center=F,scale=T)

colnames<-c(rep('blue',1),rep('red',2))

anno<-data.frame(status=factor(colnames,labels=c("K","R")))

rownames(anno)=colnames(heat_data)

color = colorRampPalette(c("navy", "white", "firebrick3"))(50)

status =c("blue", "red")

names(status)= c("K","R")

ann_colors = list(status= status)

pheatmap(heat_data,clustering_method="average",clustering_distance_rows="euclidean",clustering_distance_cols="euclidean",annotation=anno,annotation_colors=ann_colors)

KNZ_list<-read.table("K_NZ.txt",sep="\t",header=F)

diff_exp<-expres[expres[,1]%in%as.character(KNZ_list[,1]),c(1,5,6,9)]

write.table(diff_exp,"diff_exp_KNZ.txt",quote=F,sep="\t",row.names=T,col.names=T)

heat_data<-as.matrix(diff_exp[,c(2:4)])

heat_data=apply(heat_data,2,as.numeric)

heat_data=scale(heat_data,center=F,scale=T)

colnames<-c(rep('blue',1),rep('red',2))

anno<-data.frame(status=factor(colnames,labels=c("K","NZ")))

rownames(anno)=colnames(heat_data)

color = colorRampPalette(c("navy", "white", "firebrick3"))(50)

status =c("blue", "red")

names(status)= c("K","NZ")

ann_colors = list(status= status)

pheatmap(heat_data,clustering_method="average",clustering_distance_rows="euclidean",clustering_distance_cols="euclidean",annotation=anno,annotation_colors=ann_colors)

**Part Two DE lncRNA profiles (R)**

setwd("/Users/guyue/Desktop/lnc_mir/ceRNA/diff_lnc/")

expres_R1<-read.table("diff_R1.txt",sep="\t",header=T,stringsAsFactors=F,comment.char = "")

expres_R2<-read.table("diff_R2.txt",sep="\t",header=T,stringsAsFactors=F,comment.char = "")

expres_N<-read.table("diff_N.txt",sep="\t",header=T,stringsAsFactors=F,comment.char = "")

expres_Z<-read.table("diff_Z.txt",sep="\t",header=T,stringsAsFactors=F,comment.char = "")

diff_kr<-intersect(expres_R1[,1],expres_R2[,1])

diff_knz<-intersect(expres_N[,1],expres_Z[,1])

write.table(diff_kr,"diff_KR_lnc.txt",quote=F,sep="\t",row.names=F,col.names=F)

write.table(diff_knz,"diff_KNZ_lnc.txt",quote=F,sep="\t",row.names=F,col.names=F)

lncexp_R1<-unique(expres_R1[expres_R1[,1]%in%diff_kr,c(1:3)])

lncexp_R2<-unique(expres_R2[expres_R2[,1]%in%diff_kr,c(1:3)])

lncexp_R<-cbind(lncexp_R1,lncexp_R2)

lncexp_R<-lncexp_R[,-c(3,4)]

heat_data<-as.matrix(lncexp_R[,c(2:4)])

heat_data=apply(heat_data,2,as.numeric)

heat_data=scale(heat_data,center=F,scale=T)

colnames<-c(rep('red',2),rep('blue',1))

anno<-data.frame(status=factor(colnames,labels=c("R","K")))

rownames(anno)=colnames(heat_data)

color = colorRampPalette(c("navy", "white", "firebrick3"))(50)

status =c("blue", "red")

names(status)= c("K","R")

ann_colors = list(status= status)

pheatmap(heat_data,clustering_method="average",clustering_distance_rows="euclidean",clustering_distance_cols="euclidean",annotation=anno,annotation_colors=ann_colors)

lnc_mRNA_R1<-expres_R1[expres_R1[,1]%in%diff_kr,]

write.table(lncexp_R,"diff_KR_lnc_exp.txt",quote=F,sep="\t",row.names=F,col.names=T)

write.table(lnc_mRNA_R1[,c(1,5)],"KR_lnc_mRNA.txt",quote=F,sep="\t",row.names=F,col.names=T)

node_KR<-read.table("netKR.txt",sep="\t",header=F,stringsAsFactors=F,comment.char = "")

lnc_mRNA_KR<-lnc_mRNA_R1[,c(1,5)]

lnc_mRNA_KR1<-merge(lnc_mRNA_KR, node_KR, by = intersect(names(lnc_mRNA_KR[,1]), names(node_KR[,1])))

**Part Three LMP Network construction (R)**

KR_LMN<-read.table("KR_LMN.txt",sep="\t",stringsAsFactors=F)

KR_LMP<-read.table("KR_LMP.txt",sep="\t",stringsAsFactors=F)

library(igraph)

all<-rbind(KR_LMN,KR_LMP)

g<-graph(c(as.character(all[,1]),as.character(all[,2])),directed=F)

page.rank (g, algo = c("prpack", "arpack", "power"),

vids = V(g), directed = FALSE, damping = 0.85,

personalized = NULL, weights = NULL, options = NULL)

score<-page.rank(g)$vector

write.table(score,"KRnet_score.txt",quote=F,sep="\t",row.names=T,col.names=F)

net_score<-read.table("KRnet_score.txt",sep="\t",stringsAsFactors=F)

mRNA_score<-net_score[net_score[,1]%in%KR_LMN[,2],]

write.table(mRNA_score,"KRmRNA__score.txt",quote=F,sep="\t",row.names=T,col.names=F)

key_mRNA<-mRNA_score[which(mRNA_score[,2]>mean(net_score[,2])),]

hist(mRNA_score[,2],xlab = "Weight",col = "yellow",border = "blue",main="mRNA_score")

abline(v=mean(net_score[,2]),lwd=2,col="red")

write.table(key_mRNA,"key_mRNA_KR.txt",quote=F,sep="\t",row.names=F,col.names=F)

setwd("/Users/guyue/Desktop/lnc_mir/ceRNA/diff_lnc/NZ/")

KNZ_LMN<-read.table("KNZ_LMN.txt",sep="\t",stringsAsFactors=F)

KNZ_LMP<-read.table("KNZ_LMP.txt",sep="\t",stringsAsFactors=F)

library(igraph)

all<-rbind(KNZ_LMN,KNZ_LMP)

g<-graph(c(as.character(all[,1]),as.character(all[,2])),directed=F)

page.rank (g, algo = c("prpack", "arpack", "power"),

vids = V(g), directed = FALSE, damping = 0.85,

personalized = NULL, weights = NULL, options = NULL)

score<-page.rank(g)$vector

write.table(score,"KNZnet_score.txt",quote=F,sep="\t",row.names=T,col.names=F)

net_score<-read.table("KNZnet_score.txt",sep="\t",stringsAsFactors=F)

mRNA_score<-net_score[net_score[,1]%in%KNZ_LMN[,2],]

write.table(mRNA_score,"KNZmRNA__score.txt",quote=F,sep="\t",row.names=T,col.names=F)

key_mRNA<-mRNA_score[which(mRNA_score[,2]>mean(net_score[,2])),]

hist(mRNA_score[,2],xlab = "Weight",col = "yellow",border = "blue",main="mRNA_score")

abline(v=mean(net_score[,2]),lwd=2,col="red")

write.table(key_mRNA,"key_mRNA_KNZ.txt",quote=F,sep="\t",row.names=F,col.names=F)

**Part Four Enrichment analysis (R)**

library(ggplot2)

library(Cairo)

pathway=read.table("key_mRNA_enrich.txt",header=T,sep="\t")

ggplot(pathway,aes(GeneRatio,Description))+

geom_point(aes(size=Number,color=-1*LogP))+

scale_colour_gradient(low="green",high="red")+

labs(color=expression(-LogP),size="Gene number",x="GeneRatio",

title="The Enriched Function in KR")+

theme_bw()+theme(text = element_text(size = 20))+

theme(axis.text.x = element_text(size = 15))+

theme(axis.text.y = element_text(size = 15))+

theme(legend.text = element_text(size = 15))+

theme(plot.margin=unit(c(1,1,1,1),"cm"))

library(ggplot2)

library(Cairo)

pathway=read.table("key_mRNA_enrich.txt",header=T,sep="\t")

ggplot(pathway,aes(GeneRatio,Description))+

geom_point(aes(size=Number,color=-1*LogP))+

scale_colour_gradient(low="green",high="red")+

labs(color=expression(-LogP),size="Gene number",x="GeneRatio",

title="The Enriched Function in KR")+

theme_bw()+theme(text = element_text(size = 20))+

theme(axis.text.x = element_text(size = 15))+

theme(axis.text.y = element_text(size = 15))+

theme(legend.text = element_text(size = 15))+

theme(plot.margin=unit(c(1,1,1,1),"cm"))

**Part Five Visualization (R)**

lncexp_N<-unique(expres_N[expres_N[,1]%in%diff_knz,c(1:3)])

lncexp_Z<-unique(expres_Z[expres_Z[,1]%in%diff_knz,c(1:3)])

lncexp_NZ<-cbind(lncexp_N,lncexp_Z)

lncexp_NZ<-lncexp_NZ[,-c(3,4)]

heat_data<-as.matrix(lncexp_NZ[,c(2:4)])

heat_data=apply(heat_data,2,as.numeric)

heat_data=scale(heat_data,center=F,scale=T)

colnames<-c(rep('red',2),rep('blue',1))

anno<-data.frame(status=factor(colnames,labels=c("NZ","K")))

rownames(anno)=colnames(heat_data)

color = colorRampPalette(c("navy", "white", "firebrick3"))(50)

status =c("blue", "red")

names(status)= c("K","NZ")

ann_colors = list(status= status)

pheatmap(heat_data,clustering_method="average",clustering_distance_rows="euclidean",clustering_distance_cols="euclidean",annotation=anno,annotation_colors=ann_colors)

lnc_mRNA_NZ<-expres_N[expres_N[,1]%in%diff_knz,]

write.table(lncexp_NZ,"diff_KNZ_lnc_exp.txt",quote=F,sep="\t",row.names=F,col.names=T)

write.table(lnc_mRNA_NZ[,c(1,5)],"KNZ_lnc_mRNA.txt",quote=F,sep="\t",row.names=F,col.names=T)

**Part Six ceRNA construction (Perl)**

use strict;

use warnings;

my %hash=();

open(RF,"diff_lncRNA.txt") or die $!;

while(my $line=<RF>){ chomp($line); $hash{$line}=1;}

close(RF);

open(RF,"mircode.txt") or die $!;

open(WF,">lncRNA_mircode.txt") or die $!;

while(my $line=<RF>){ if($.==1){print WF $line; next;}

my @arr=split(/\t/,$line);

my @zeroArr=split(/\|/,$arr[1]);

if(exists $hash{$zeroArr[0]}){print WF $line;}}

close(WF);

close(RF);

###lncRNA_miRNA

use strict;

use warnings;

my %hash=();

open(RF,"diff_miRNA.txt") or die $!;

while(my $line=<RF>){ chomp($line); $line=~s/^\s+|\s+$//g; $hash{$line}=1;}

close(RF);

my %repHash=();

open(RF,"lncRNA_mircode.txt") or die $!;

open(WF,">lncRNA_miRNA.txt") or die $!;

print WF "lncRNA\tmiRNA\n";

while(my $line=<RF>){ my @arr=split(/\t/,$line);my @threeArr=split(/\//,$arr[3]); $threeArr[0]=~s/miR\-//g; foreach my $mirna(@threeArr){ if(exists $hash{"hsa-mir-$mirna"}){unless(exists $repHash{"$arr[1]\thsa-mir-$mirna"}){print WF "$arr[1]\thsa-mir-$mirna\n"; $repHash{"$arr[1]\thsa-mir-$mirna"}=1;}}}}

close(WF);

close(RF);

###miRNA_target

use strict;

use warnings;

my %miHash=();

open(RF,"miRNA.txt") or die $!;

while(my $line=<RF>){ chomp($line); $line=~s/^\s+|\s+$//g; $miHash{$line}=1;}

close(RF);

my %hash=();

my @files=glob("*.tsv");

my @dbs=();

foreach my $file(@files){my $db=$file; $db=~s/\.tsv//g; push(@dbs,$db);

open(RF,"$file") or die $!;

while(my $line=<RF>){ chomp($line); my @arr=split(/\t/,$line); if(exists $miHash{$arr[0]}){ my $mirnaGene="$arr[0]\t$arr[1]"; ${$hash{$mirnaGene}}{$db}=1;}}close(RF);}

open(WF,">result.xls") or die $!;

print WF "miRNA\tGene\t" . join("\t",@dbs) . "\tSum\n";

foreach my $key(keys %hash){my $outLine=$key; my $sum=0; foreach my $db(@dbs){if(exists ${$hash{$key}}{$db}){ $sum++; $outLine=$outLine . "\t1";} else{$outLine=$outLine . "\t0"; }}

if($sum>=3){ print WF $outLine . "\t$sum\n"; }

}

close(WF);

###ceRNA###

use strict;

use warnings;

my %hash=();

open(RF,"DEmRNA.txt") or die $!;

while(my $line=<RF>){ chomp($line); $hash{$line}=1;}

close(RF);

open(RF,"miRNA_Target.txt") or die $!;

open(WF,">miRNA_mRNA.txt") or die $!;

while(my $line=<RF>){ if($.==1){print WF $line;next;}

my @arr=split(/\t/,$line);

my @zeroArr=split(/\|/,$arr[1]);

if(exists $hash{$zeroArr[0]}){print WF $line;}}

close(WF);

close(RF);
